# Participant views on practical considerations for feedback of individual genetic research results: a case study from Botswana

**DOI:** 10.1080/11287462.2023.2192329

**Published:** 2023-04-12

**Authors:** Dimpho Ralefala, Mary Kasule, Olivia P. Matshabane, Ambroise Wonkam, Mogomotsi Matshaba, Jantina de Vries

**Affiliations:** a Faculty of Health Sciences, Department of Medicine, University of Cape Town, Cape Town, South Africa; b Office of Research and Development, University of Botswana, Gaborone, Botswana; c Faculty of Health Sciences and Groote Schuur Hospital, Cape Town, South Africa; d Botswana-Baylor Children’s Clinical Centre of Excellence, Gaborone, Botswana; e Baylor College of Medicine, Houston, TX, USA

**Keywords:** Practical considerations, feedback of results, genetics, parents, adolescents, Botswana, Africa

## Abstract

Key to discussions around feedback of individual results from genomics research are practical questions on how such results should be fed back, by who and when. However, there has been virtually no work investigating these practical considerations for feedback of individual genetic results in the context of low-and middle-income countries (LMICs), especially in Africa. Consequently, we conducted deliberative focus group discussions with 6 groups of adolescents (*n* = 44) who previously participated in a genomics study in Botswana as well as 6 groups of parents and caregivers (*n* = 49) of children who participated in the same study. We also conducted in-depth interviews with 6 adolescents and 6 parents or caregivers. Our findings revealed that both adolescents and parents would prefer to receive their individual genetic results in person, with adolescents preferring researchers to provide feedback, while parents preferred doctors who are associated with the study. Both adolescents and parents further expressed that feedback should be supported by counselling but differed on the timing of feedback, with preferences ranging from feedback as quickly as possible to feedback at project end. In conclusion, decisions on practicalities for feedback of results should be done in account of participants’ context and considerations of participants’ preferences.

## Background

Whilst there is a growing body of critical scholarship investigating ethical challenges in African genomics research, the majority of such work focuses on a small number of challenges relating for instance to informed consent (Marshall et al., 2014; Munung et al., 2016; Tindana et al., 2012; Tindana & de Vries, 2016) and community engagement (Campbell et al., 2015; Folayan et al., 2015; Rotimi et al., 2007; Tindana et al., 2015). However, equally important to ethical deliberations around genomics research are questions on how individual results from genomic studies should be fed back, by who and when. While there is a developing consensus that some research results ought to be fed back, what remains unclear is specifically with whom this obligation lies. Generally, no individual is identified specifically to perform this task (Knoppers et al., 2006).

Most literature on this topic is from high-income countries (HICs). Nonetheless, these studies have generated varying results on delivery preferences for returning different types of genetic and genome sequencing results. With regards to sources of information, previous research on feedback of genetic results has suggested preferences for different types of clinicians. For example, findings from a US-based focus group discussion with some members of the general public, identified that doctors or physicians were mostly preferred, followed by genetic counsellors, and then psychiatrists or therapists (Yu, Crouch, et al., 2014). Similarly, other studies from the US which specifically address feedback of genetic results have also yielded varied findings, with preferences for results to be returned in-person, via the internet, mail, phone or through a combination of these techniques (Bui et al., 2014; Levenseller et al., 2014; Wright et al., 2014; Yu et al., 2013; Yu, Harrell, et al., 2014). Few studies have explored patients preferences for when they would like to have their genetic results fed back, although concerns have been raised that patients might not be emotionally ready to find out about secondary genetic results at an initial disclosure meeting (Kaphingst et al., 2016).

There has been virtually no work investigating these practical considerations for feedback of individual results from genomics research in the context of low-and middle-income countries (LMICs), particularly in Africa, despite increasing genomic research in these settings. As a result, there is currently no evidence for how individual genetic research results can feasibly be fed back, nor is there practical insight into how participants expect feedback to be given – both of which are essential to the development of a feedback practice in African genomics research. In order to contribute to this discussion, we undertook an empirical study to explore participants’ preferences for feedback of individual results in an HIV-TB genomic research project in Botswana. Specifically, we sought to generate an evidence base regarding participants’ preferences on how individual results from genomic studies should be fed back, by who and when.

## Methods

### Ethical review

Ethics approval for this study was obtained from the Faculty of Health Sciences Health Research Ethics Committee (HREC REF: 010/2019) at the University of Cape Town, the University of Botswana Institutional Review Board (UBR/RES/IRB/BIO/118), the Health Research and Development Committee (HPDME: 13/18/1) of the Ministry of Health and Wellness in Botswana and the Botswana-Baylor Children’s Clinical Centre of Excellence IRB. Written assent was obtained from all adolescent participants along with written consent by their parents or caregivers. Parents and caregivers also provided signed informed consent for their own participation in the study.

### Study population

This study was conducted in Botswana, an upper middle-income country in Southern Africa, with a Gross Domestic Product per capita of 7080.12 United States Dollars (USD) (Botswana, 2018). In 2014, the country spent about 5.4 percent of its GDP on health expenditure (WHO, 2016) for an estimated population of over 2 million people. This is reflected by an extensive health-care system across the country including hospitals, clinics, health posts and mobile stops spread across 27 health districts. Health care (inclusive of hospital care, laboratory tests and medications) is provided free-of-charge in public sector facilities for all citizens and almost 95 percent of Botswana’s population lives within 8 kilometres of the nearest health facility (Botswana, 2015). Although, private healthcare is also available in Botswana, about 98 percent of all health facilities are operated by the government (Tapera et al., 2018). Nationally, most out-patients services are offered through a network of clinics. In 2009, 66 percent of outpatients were attended in clinics, 21 percent at health posts, 7 percent at hospitals while only 6 percent were attended at primary hospitals. This pattern is in line with the government’s policy of promoting the utilisation of clinics and health post services before visiting hospitals. As a result clinics and health posts are spread across the country, and are the most accessible of all types of health facilities (Botswana, 2010). As of 2009, Botswana’s public healthcare system had a total of 819 professional doctors, 5816 professional nurses, 52 Social Workers, 13 Social Workers Technicians, and 7 psychologists among other professionals (Botswana, 2010). It is through this system that patients are provided with a comprehensive care and treatment of common problems (Tapera et al., 2018).

Nonetheless, our study was conducted at Botswana-Baylor Children’s Clinical Centre of Excellence, which is a public-private partnership between the government of Botswana and Baylor College of Medicine’s Baylor International Paediatric AIDS Initiative. Botswana-Baylor Children's Clinical Centre of Excellence is a paediatric and adolescent clinic specialising in HIV treatment. As such, it is the main care unit for children, adolescents, and young adults with HIV and has been caring for these patients for almost two decades. The BBCCCE also conducts research, including a genomic study which is part of the Collaborative African Genomics Network (CAfGEN), which studies genes of children and adolescents with HIV and TB, to inform the advancement of new treatments to prevent or supress these infections (BBCCCE, 2020). It is from the CAfGEN study that our participants were recruited. The CAfGEN study was chosen as a case study because it is one of the few genomic studies being carried out in Botswana and presented an opportunity to explore issues related to feedback of individual results in genomics research. Consequently, adolescents enrolled in the CAfGEN study, who participated in our study, also receive medical care from BBCCCE. In our study, we specifically recruited adolescents aged 15–18 years as well as parents and caregivers of children and adolescents aged 2–18 years. We purposively enrolled 93 participants; 44 adolescents who previously participated in the CAfGEN study and 49 parents and caregivers of children and adolescents who participated in the same study.

### Data collection procedures

Participants were recruited in person during their visits to the BBCCCE as well as over the telephone where necessary. Recruitment was done by the CAfGEN study nurse and research assistant who briefed participants about our study and relayed those interested to us, for the consent process and enrolment into the study.

In our study, we used deliberative focus group discussions (dFGDs) (Rothwell et al., 2016) and in-depth interviews (IDIs) to explore a wide range of issues relating to the feedback of individual genetic research results including participants’ views on how and when they would like to receive results and by whom. A dFGD manual with detailed instructions was developed to guide the discussions. This was piloted with 2 groups of parents and caregivers, as well as 1 group of adolescents, while the IDI tool was piloted with 3 parents/caregivers and 2 adolescents.

The deliberative focus group discussions method used for this study, is a combination of a traditional FGD method with extended opportunities for learning and discussion, in an effort to allow participants to engage with the topic under discussion in more depth. The dFGD approach used in this study involved an interactive information-sharing session with participants which lasted about 45–60 minutes. Case studies elaborating particular ethical considerations in the return of individual genetic results were used for information sharing during the first meeting. This allowed participants to learn about genes, their functions and impact on health. After a short break, this was followed by a discussion to get participants’ immediate thoughts about feedback of results from genomics research. This session lasted about 60–90 minutes. At the end of this meeting, participants were given an information leaflet covering basic information on genetics as a reference to help them reflect on the information and issues discussed during the meeting. They were later invited for a second dFGD meeting a week later, where their opinions about feedback of findings was interrogated further. This second dFGD took about 30–60 minutes. Data were collected until saturation was reached. Data saturation was discussed by DR, MK and JDV. dFGD meetings were held with 12 groups of participants (6 groups of adolescents and 6 groups of parents and caregivers), resulting in 24 dFGD meetings in total. These were complemented with 12 in-depth interviews (6 adolescents and 6 parents/caregivers) with people who had also participated in the dFGDs. The IDIs focused on probing some of the early insights from the study. These were conducted with participants from the dFGDs who either engaged more in the discussions or held uncommon views, as well as a few others who were less interactive in the dFGDs so as to give them an opportunity to share their views in a more private conversation. However, no strict criteria were used to assess participants’ level of engagement in the dFGDs. IDI sessions took 30–80 minutes. The dFGDs and IDIs for parents and caregivers were conducted in Setswana using translated tools, while a combination of Setswana and English was used in the dFGDs and IDIs with adolescents. The choice of language for discussions was decided by participants. Data were collected from February 2019 to March 2020.

### Participants’ demographics

The majority of adolescents (61 percent) in our study were female. Adolescents in our study were aged between 15–18 years; with 18 percent aged 15 years, 36 percent aged 16 years, 25 percent aged 17 years, 16 percent were 18 years old, and the rest (5 percent) did not record their age. Most adolescents (93 percent) were either attending or had completed junior school or high school. Half of the adolescents lived in villages surrounding the city of Gaborone where this study was based, 39 percent lived in the city while 11 percent did not report their place of residence. Almost all parents and caregivers (92 percent) were female. One likely reason for this could be that women are usually the primary caregivers in Botswana (Kang'ethe, 2011; 2013; Maundeni et al., 2009) owing to sociocultural and legal factors (Jorosi-Tshiamo et al., 2013). Majority of parents and caregivers (39 percent) were aged between 41 and 50 years and 94 percent had educational qualifications ranging from primary to tertiary education. Similar to the adolescent study population, most parents and caregivers (57 percent) resided in villages surrounding the city of Gaborone while 43 percent lived in the city.

### Data analysis procedures

All discussions were audio-recorded, transcribed, translated into English and imported into NVivo qualitative data analysis software Version 12 for coding and data analysis. Data were transcribed by a Research Assistant and verified by the primary author (DR), both being native Setswana speakers and proficient in English. We applied both an inductive and deductive approach to coding. The Framework Method for data analysis was used to guide the analysis of the data (Gale et al., 2013). This followed five key stages that are closely aligned with the analytical process of thematic analysis, and which involve: (a) familiarisation with the data (b) establishing a thematic framework (c) indexing (d) charting (e) data interpretation (Ritchie et al., 1994). See Ralefala et al. (2020), for more information on the methodological approach used in this study.

## Results

When deciding on which results should be returned, a key question relates to practical aspects of how results should be fed back, by whom and when. Decisions in relation to these practical questions impact on the feasibility and cost of returning individual genetic research results – for instance, if the preference is for telephonic consults then this would be more cost-effective than in-person consultations. In this paper, we specifically report on our research participants’ preferences for these practical elements of returning results. Although we also discussed other issues, including those related to feasibility of feedback of results and cost for the study as well as actionability of results, these findings have not been presented in this paper.

### Participant views on how to feedback and by whom

Our findings revealed that most adolescents expressed that researchers should feedback results because they are the ones who conducted the study and are more knowledgeable about genes and their outcomes. In addition, adolescents described that over the course of their participation in a research project, they developed a relationship with the research team, and they considered that it would make sense for them to receive information about the study from those team members. However, there were also divergent views with some other adolescents recommending that a treating doctor should be the one to feedback results because they know more about the health of the research participant and could give them further information on what to do. Only one adolescent shared a view that a counsellor should feedback results.

By contrast, most parents and caregivers expressed that researchers should relay results to a doctor who would then give them to the participant. Their view was that a doctor is better placed to advise the participant on their condition in light of their medical history and advice on steps or precautions to be taken. Yet in this case also, parents seem to be referring to medical staff who are linked to the study in some way. This is evidenced by the statement below from one of the parents:

*The researcher could tell those that they would have been working with, if it’s a doctor so that they inform the participant about the results of the research, the medical staff could be the ones giving back the results because they would know how to handle such cases. (IDI-P005)*

Another parent said:

*As the researcher you will give the findings back to the partner you are working with, just like you have partnered with Baylor clinic, so you will give Baylor the findings and they will know how to contact their clients and give them their results. (P4G5)*

As seen from above quote, this view seems to be influenced by the setup at Botswana-Baylor Children’ s Clinical Centre of Excellence which is a public-private paediatric and adolescent clinic specialising in HIV treatment. As such, it is the main care unit for patients with HIV and has been caring for these patients for almost two decades. Yet the BBCCCE also conducts research, including the CAfGEN study in which (the children of) all of our research participants had participated. In the BBCCCE, adolescent patients are seen regularly by doctors who are often well-acquainted with the participant’s medical history. Against that setting, feeding back individual genetic research results through the BBCCCE could make sense. However, patients in the national health care system do not have regular doctors as doctors work on rotation, making it difficult for the suggested arrangement to be feasible. Similarly, other genomic research projects that are not embedded within a clinical care programme also may not have the kind of clinical relationship with patients that would enable the return of individual genetic research results.

In addition to preferring results being shared by the BBCCCE doctor, some parents expressed that feedback of results should rather be done by a team comprising of the researcher, doctor and counsellor, while others shared that this responsibility should be assigned to a counsellor. Only a few parents shared that the researchers should be the ones to feedback. In this context, a researcher was described to participants as a scientist who may not have a medical background, for example a geneticist. This explanation was given to help participants differentiate between doctors and researchers.

All the adolescents expressed that they would prefer to have their genetic results fed back in person (face-to-face), so that they could receive counselling if they need it. They shared that if researchers “ … use any other method, the information could get lost” (A2G3). Similarly, parents expressed that they would prefer to be given their results in person so that they could be given counselling.

Although most adolescents initially expressed that results should be fed back by the researchers, they additionally pointed out that there should be further counselling for feedback of results done by a counsellor, with only a few indicating preference for their mother, a social worker or a psychologist. One adolescent emphasised the importance of counselling by saying:

* … I think it would help if participants were sat down and spoken to rather than just giving them their results and have them be on their way. There are good listeners who will benefit from comforting counselling sessions on issues like if I have this gene I shouldn’t lose hope … (IDI-A001)*

Other adolescents also expressed that a counselling session could provide an opportunity for them to be given more information about the genetic condition as well as how to take care of themselves so that the genetic condition does not affect them. They also shared that going through counselling could clarify that having a gene does not mean one is already ill, but that there is a possibility of having that condition in future, comfort them and help them accept their condition. When probed about what kind of information participants would need to make sense of individual genetic findings in genomic research, adolescents expressed that they would like to know as much information related to genes as possible. One adolescent shared that “ … researchers should give more information on what a gene is and what it means to have a gene of a certain illness and how to prevent and treat that illness” (IDI-A002). Others indicated that participants should be taught about different genetic illnesses and whether they are preventable or not and that participants should be counselled on how to “ … handle the situation when they receive bad results … (IDI-A006)”, keep a positive mind and not worry much about what is going to happen in their future.

Similar to adolescents, whilst most parents and caregivers think that doctors should do the actual feeding back, there are also many parents and caregivers who think that this should be done in conjunction with counselling support. When asked about who should provide counselling for feedback of results, one parent stated that:

*It will be the counselling team, in most cases people are not trained to be counsellors, even the doctors don’t have the counselling skills, they just know the medicine so, that one I would say participants should be taken straight to the trained professionals. (IDI-P004)*

One other participant mentioned that a counsellor could help them emotionally while a pastor could help to prepare them spiritually. Parents and caregivers further pointed out that providing counselling as part of the feedback process is very important in helping participants accept their situation. They shared that as part of counselling, participants should be provided with information about the discovered mutation. Parents additionally mentioned the need for pre-counselling to prepare the participants to receive their results, but also highlighted that counselling should be a step-by-step process that should continue after feedback of results so as to guide participants on what they could do to manage the mutation from developing into an illness. One parent suggested that:

*They sit down with me and counsel me about the possible outcomes of the results and that whatever outcome, there could be help, so as to calm my emotions down. I don’t think you can be told such results without any counselling. They should tell you that although the study was looking for this and that, we also found some other things that we did not expect. One strategy is to ask the participant how they would receive the results if anything else were to be found in their blood sample … (P12G5)*

In addition, parents were also probed about what kind of information participants would need to make sense of individual genetic findings in genomic research and one parent shared that “ … a lot of information about genes should be given..[…]..you should teach people about the different genetic illnesses so that people don’t assume that they are bewitched or whatever” (IDI-P001). Another shared that in returning results, “ … researchers could start of by teaching people about illnesses that pass from one person to the next in the family due to genes” (IDI-P003). Additionally, some parents shared that researchers could:

* … cover that genetic studies can unearth some illnesses that you didn’t know you had, that way you could have a resolution to the situation, and I would also have that important information that explains that having a certain gene of a particular illness doesn’t mean that you have that illness, but it could develop into an illness at the end. (IDI-P005)*

Overall, there seemed to be an expectation by both adolescents, parents and caregivers for the research team to make provision for counselling support by the trained professionals, who were mostly identified as counsellors. However, in this context, participants were not probed on whether counsellors would need to be trained in genetics or not.

### Participants views on when to feedback

Our findings revealed that adolescents generally would like to receive their results at the end of the study. They reasoned that this would allow researchers ample time to do their investigations. However, others were concerned that delaying feedback may not be ideal as some genetic conditions are life-threatening hence waiting till the end of the study may be too late. To resolve this, one adolescent suggested that “if the results are not that life threatening you can wait before telling her, if the results are very risky then the researchers should tell the participant right away” (A2G6). On the contrary, most parents would prefer to be given results as early as possible and not to wait for the study to be completed. Their concern was that waiting for the completion of the study would be too late and that timely feedback could enable them to look for the necessary help, should there be any. Nonetheless, one parent shared that “researchers shouldn’t be rushed because their job is complex; they should be left to do their job thoroughly, even if it takes them 3 years” (P2G6). This view was shared by other parents. Similar to adolescents’ discussions, these conflicting views were mediated by one parent who suggested that:

* … it will depend on the kind of the illness, if it is an illness that requires immediate attention then they should let you know in a timely manner, but if it is something that you can live with and have it not affect you, then they should continue with their study and give results at the end.*

## Discussion

Our study revealed that almost all the adolescents and parents would prefer to have their results fed back in person. This is consistent with views expressed by participants in Kaphingst et al. (2016) and O'Daniel and Haga (2011). Participants shared that a face-to-face feedback meeting provides an opportunity for counselling. Whilst most adolescents in our study would prefer to have these results fed back by researchers, parents would rather have researchers work with doctors to relay the results to participants. This is because they thought that doctors are better placed to advise participants on their condition, taking note of their medical history, and providing guidance on risk management and follow-up steps. Such views were also expressed by some participants in Yu, Crouch, et al. (2014) who would prefer to have their results fed back by healthcare providers, so that they could assist them to understand results in consideration of their current health status and by offering counselling support. Additionally, a majority (42%) of participants in O'Daniel and Haga (2011) also chose to learn their results from their physician.

In our study, the parents did not seem to refer to a family physician, but rather to doctors associated with the clinical programme at BBCCCE. At this centre, patients have regular doctors who are well-acquainted with their patients’ medical history. This is not the case in the national health care system, especially in main hospitals where doctors rotate and where electronic health records are not used consistently, making it difficult for doctors to be well acquainted with their patients’ medical background. This was also identified as a limitation for most African settings by Wonkam and de Vries (2020).

Although participants indicated wanting results fed back by researchers or medical staff, both adolescents and parents expressed that feedback should be supported by counselling. Counselling is expected to provide participants with background genetic information to help participants understand their situation as well as information that could help them cope. This is in line with a recommendation by O'Daniel and Haga (2011), who shared that the feedback of these results could imitate a genetic counselling session involving an overview of genetics as well as the specifics of the study and limitations for interpreting the results. Again, the feasibility of implementing this through the national health care system in Botswana is questionable due to lack of genetic counsellors in the country. Although most major hospitals in Botswana’ would normally have a psychologist or a social worker or both depending on the size of the hospital, they are not usually trained in genetics counselling. This is true even in a setup like BBCCCE where there are both a counsellor and a clinical psychologist whom patients may be referred to. As a result, projects intending to feedback genetic research results may need to work with these professionals and train them in the basic principles of genetics as well as the complexities of genetic results for them to provide meaningful feedback to participants. Elsewhere, it has been suggested that nurses could also be trained to provide basic genetic counselling in addition to their current roles (Barr et al., 2018; Nembaware et al., 2019). This is because nurses are usually at the forefront of many healthcare settings in Africa and often have comprehensive information about patients, families, as well as communities (Prows et al., 2005). Nonetheless, where taking advantage of available professionals to provide genetic counselling is not possible due to limited resources in the public healthcare system, researchers may need to make arrangements to provide such services through budgeting to employ a counsellor who will be trained to assist with the return of results at project end. In this case, the obligation for researchers to provide ancillary care to participants in the form of counselling support, may be stronger. According to Richardson (2008), in doing research, researchers often take on some responsibility with regard to the health of research participants as a result of having entered into a researcher-participant relationship. The nature of this relationship is such that researchers have ancillary care obligations for findings that are related to the health condition being studied. As further highlighted by Richardson and Belsky (2004), this obligation may be strengthened by participants’ degree of vulnerability and the extent to which the duty to rescue reinforces these grounds.

Lastly, our findings revealed that both adolescents and parents described preferences for the timing of feedback, ranging from feedback as soon as possible to feedback at project end. Most adolescents were more inclined to having feedback of results at the end of the study as compared to parents who preferred to have results fed back as soon as possible. Participants who preferred to have results fed back immediately argued that waiting for the completion of the study could be too late for certain serious and urgent genetic conditions that might need immediate action. These views are consistent with findings from the study by Kaphingst et al. (2016) which revealed that many participants preferred to receive their sequencing results within the shortest time possible. While a few suggested for feedback of results to be staged, to provide immediate feedback for results that need urgent attention only, and have the rest fed back at the end of the study. According to participants in Yu, Crouch, et al. (2014), prioritising and staging return of results could help in avoiding information overload.

## Conclusions

In conclusion, our participants preferred that feedback of individual genetic results should be done in person. Decisions about who to feedback and counsel participants are project-dependent and should be done in account of study setting. For example, in settings like BBCCCE it might be feasible for the study team to relay participants’ results to treating doctors in the same centre to feedback to participants, while also organising a counsellor for counselling if necessary. However, in cases where a study is done in a public facility with limited resources, that could be difficult to implement. Consequently, researchers may have to take up the responsibility of feeding back individual results as well as providing genetic counselling in such settings. To make these decisions, researchers should familiarise themselves with their research settings up-front and engage with relevant stakeholders so as to make informed decisions regarding the feasibility and acceptability of their approach to feedback of results. Our participants also suggested that staggered feedback could be considered, where results that require urgent follow-up are fed back as soon as possible while others are fed back when the project ends.

## Limitations

Although these findings may not be generalised to other contexts, they are important to the literature on feedback of results in genomics research in Botswana. One limitation is that, most of parents and caregivers in our study (92%) were female, possibly because women are usually the primary caregivers in Botswana (Kang'ethe, 2011; Kang’ethe, 2013; Maundeni et al., 2009) owing to sociocultural and legal factors (Jorosi-Tshiamo et al., 2013). As a result, this means that the views of male participants were not well represented in this study. Secondly, our participants’ experience of health care provision might be different from others because they have access to free state-of-the-art health care at BBCCCE which is different from elsewhere in the country. Thirdly, although we took care during the dFGD process to share information about the complexity of genetic results, this still seemed quite vague for participants as the feedback of individual genetic research results was presented as a hypothetical situation. As a result, participants’ views and preferences might change when presented with a real-life situation. Additionally, the dFGD approach used in this study is novel and allows for information to be shared and for participants to be engaged for a longer timeframe which means they arguably become more comfortable to share their views as compared to normal FGDs. This could be an effective way to engage participants on complex concepts such as genetics, but there is a risk that the material presented in the course of the discussion bias participants to articulate or support particular views.

## Data Availability

The datasets used and/or analysed during the current study are available from the corresponding author on reasonable request.

## References

[CIT0001] Barr, J. A., Tsai, L. P., Welch, A., Faradz, S. M., Lane-Krebs, K., Howie, V., & Hillman, W. (2018). Current practice for genetic counselling by nurses: An integrative review. *International Journal of Nursing Practice*, *24*(2), e12629. 10.1111/ijn.12629 29462836

[CIT0002] BBCCCE. (2020). Botswana-Baylor Children's Clinical Centre of Excellence. https://botswanabaylor.org/about.html. 10.1177/29768357251327567 PMC11951437 40162092

[CIT0003] Botswana. (2010). *Health Statistics Report* 2010. https://www.statsbots.org.bw/sites/default/files/publications/Health%20Statistics%20Report%202010.pdf.

[CIT0004] Botswana. (2015). *Botswana-Maternal Mortality Ratio* 2014. http://www.statsbots.org.bw/sites/default/files/publications/Botswana%20-%20Maternal%20%20Mortality%20Ratio%202014.pdf.

[CIT0005] Botswana. (2018). *Gross domestic product: Second quarter of 2018*. In Statistics-Botswana (Ed.): Gaborone: Statistics Botswana. http://www.statsbots.org.bw.

[CIT0006] Bui, E. T., Anderson, N. K., Kassem, L., & McMahon, F. J. (2014). Do participants in genome sequencing studies of psychiatric disorders wish to be informed of their results? A survey study. *Plos One*, *9*(7), e101111. 10.1371/journal.pone.0101111 24983240 PMC4077756

[CIT0007] Campbell, M. M., Susser, E., de Vries, J., Baldinger, A., Sibeko, G., Mndini, M. M., Mqulwana, S. G., Ntola, O. A., Ramesar, R. S., & Stein, D. J. (2015). Exploring researchers’ experiences of working with a researcher-driven, population-specific community advisory board in a South African schizophrenia genomics study. *BMC Medical Ethics*, *16*(1), 1–9. 10.1186/s12910-015-0037-5 26135122 PMC4487967

[CIT0008] Folayan, M. O., Oyedeji, K. S., & Fatusi, O. A. (2015). Community members’ engagement with and involvement in genomic research: Lessons to learn from the field. *Developing World Bioethics*, *15*(1), 1–7. 10.1111/dewb.12020 23594220

[CIT0009] Gale, N. K., Heath, G., Cameron, E., Rashid, S., & Redwood, S. (2013). Using the framework method for the analysis of qualitative data in multi-disciplinary health research. *BMC Medical Research Methodology*, *13*(1), 117. 10.1186/1471-2288-13-117 24047204 PMC3848812

[CIT0010] Jorosi-Tshiamo, W. B., Mogobe, K. D., & Mokotedi, M. T. (2013). Male involvement in child care activities: A review of the literature in Botswana. *African Journal of Reproductive Health*, *17*(4). 24558780

[CIT0011] Kang’ethe, S. (2013). Feminization of poverty in palliative care giving of people living with HIV and AIDS and other debilitating diseases in Botswana. A literature review. *Journal of Virology & Microbiology*, *2013*.

[CIT0012] Kang'ethe, S. M. (2011). Gender discrepancies in the HIV/AIDS community home-based care programme in Kanye, Botswana. *South African Family Practice*, *53*(5), 467–473. 10.1080/20786204.2011.10874136

[CIT0013] Kaphingst, K. A., Ivanovich, J., Elrick, A., Dresser, R., Matsen, C., & Goodman, M. S. (2016). How, who, and when: Preferences for delivery of genome sequencing results among women diagnosed with breast cancer at a young age. *Molecular Genetics & Genomic Medicine*, *4*(6), 684–695. 10.1002/mgg3.254 27896289 PMC5118211

[CIT0014] Knoppers, B. M., Joly, Y., Simard, J., & Durocher, F. (2006). The emergence of an ethical duty to disclose genetic research results: International perspectives. *European Journal of Human Genetics*, *14*(11), 1170–1178. 10.1038/sj.ejhg.5201690 16868560

[CIT0015] Levenseller, B. L., Soucier, D. J., Miller, V. A., Harris, D., Conway, L., & Bernhardt, B. A. (2014). Stakeholders’ opinions on the implementation of pediatric whole exome sequencing: Implications for informed consent. *Journal of Genetic Counseling*, *23*(4), 552–565. 10.1007/s10897-013-9626-y 23846343 PMC3849137

[CIT0016] Marshall, P. A., Adebamowo, C. A., Adeyemo, A. A., Ogundiran, T. O., Strenski, T., Zhou, J., & Rotimi, C. N. (2014). Voluntary participation and comprehension of informed consent in a genetic epidemiological study of breast cancer in Nigeria. *BMC Medical Ethics*, *15*(1), 38. 10.1186/1472-6939-15-38 24885380 PMC4032563

[CIT0017] Maundeni, T., Osei-Hwedie, B. Z., Mukaamambo, E., & Ntseane, P. G., (Eds.). (2009). *Male involvement in sexual and reproductive health,’ prevention of violence and HIV/AIDS in Botswana*. Made Plain Communications.

[CIT0018] Munung, N. S., Marshall, P., Campbell, M., Littler, K., Masiye, F., Ouwe-Missi-Oukem-Boyer, O., Seeley, J., Stein, D. J., Tindana, P., & de Vries, J. (2016). Obtaining informed consent for genomics research in Africa: Analysis of H3Africa consent documents. *Journal of Medical Ethics*, *42*(2), 132–137. 10.1136/medethics-2015-102796 26644426 PMC4752624

[CIT0019] Nembaware, V., African Genomic Medicine Training Initiative, & Mulder, N. (2019). The African genomic medicine training initiative (AGMT): showcasing a community and framework driven genomic medicine training for nurses in Africa. *Frontiers in Genetics*, *10*, 1209. 10.3389/fgene.2019.01209 31921282 PMC6934054

[CIT0020] O'Daniel, J., & Haga, S. B. (2011). Public perspectives on returning genetics and genomics research results. *Public Health Genomics*, *14*(6), 346–355. 10.1159/000324933 21555865 PMC3221258

[CIT0021] Prows, C. A., Glass, M., Nicol, M., Skirton, H., & Williams, J. (2005). Genomics in nursing education. *Journal of Nursing Scholarship*, *37*(3), 196–202. 10.1111/j.1547-5069.2005.00035.x 16235858

[CIT0022] Ralefala, D., Kasule, M., Wonkam, A., Matshaba, M., & de Vries, J. (2020). Do solidarity and reciprocity obligations compel African researchers to feedback individual genetic results in genomics research? *BMC Medical Ethics*, *21*(1), 1–11. 10.1186/s12910-020-00549-4 33148222 PMC7640670

[CIT0023] Richardson, H. S. (2008). Incidental findings and ancillary-care obligations. *Journal of Law, Medicine & Ethics*, *36*(2), 256–270. 10.1111/j.1748-720X.2008.00268.x PMC2575243 18547193

[CIT0024] Richardson, H. S., & Belsky, L. (2004). The ancillary-care responsibilities of medical researchers: An ethical framework for thinking about the clinical care that researchers Owe their subjects. *The Hastings Center Report*, *34*(1), 25–33. 10.2307/3528248 15098404

[CIT0025] Ritchie, J., Spencer, L., Bryman, A., & Burgess, R. (1994). Qualitative data analysis for applied policy research. *Analyzing Qualitative Data*, *173*, 194.

[CIT0026] Rothwell, E., Anderson, R., & Botkin, J. R. (2016). Deliberative discussion focus groups. *Qualitative Health Research*, *26*(6), 734–740. 10.1177/1049732315591150 26078330 PMC5847289

[CIT0027] Rotimi, C., Leppert, M., Matsuda, I., Zeng, C., Zhang, H., Adebamowo, C., Ajayi, I., Aniagwu, T., Dixon, M., Fukushima, Y., Macer, D., Marshall, P., Nkwodimmah, C., Peiffer, A., Royal, C., Suda, E., Zhao, H., Wang, V. O., McWen, J., … The International HapMap Consortium. (2007). Community engagement and informed consent in the international HapMap project. *Public Health Genomics**,* *10*(3), 186-198. 10.1159/000101761 17575464

[CIT0028] Tapera, R., Moseki, S., & January, J. (2018). The status of health promotion in Botswana. *Journal of Public Health in Africa**,* *9*(1). 10.4081/jphia.2018.699 PMC6057722 30079160

[CIT0029] Tindana, P., Bull, S., Amenga-Etego, L., de Vries, J., Aborigo, R., Koram, K., Kwiatkowski, D., & Parker, M. (2012). Seeking consent to genetic and genomic research in a rural Ghanaian setting: A qualitative study of the MalariaGEN experience. *BMC Medical Ethics*, *13*(1), 1–12. 10.1186/1472-6939-13-15 22747883 PMC3441464

[CIT0030] Tindana, P., & de Vries, J. (2016). Broad consent for genomic research and biobanking: Perspectives from Low- and middle-income countries. *Annual Review of Genomics and Human Genetics*, *17*(1), 375–393. 10.1146/annurev-genom-083115-022456 26905784

[CIT0031] Tindana, P., de Vries, J., Campbell, M., Littler, K., Seeley, J., Marshall, P., Troyer, J., Ogundipe, M., Alibu, V. P., Yakubu, A, & Parker, M. (2015). Community engagement strategies for genomic studies in Africa: A review of the literature. *BMC Medical Ethics*, *16*(1), 24. 10.1186/s12910-015-0014-z 25889051 PMC4407431

[CIT0032] WHO. (2016). *Botswna: HIV Country Profile*. https://www.who.int/hiv/data/Country_profile_Botswana.pdf?ua = 1.

[CIT0033] Wonkam, A., & de Vries, J. (2020). Returning incidental findings in African genomics research. *Nature Genetics*, *52*(1), 17–20. 10.1038/s41588-019-0542-4 31768070 PMC7255819

[CIT0034] Wright, M. F., Lewis, K. L., Fisher, T. C., Hooker, G. W., Emanuel, T. E., Biesecker, L. G., & Biesecker, B. B. (2014). Preferences for results delivery from exome sequencing/genome sequencing. *Genetics in Medicine*, *16*(6), 442–447. 10.1038/gim.2013.170 24310310 PMC4597884

[CIT0035] Yu, J. H., Crouch, J., Jamal, S. M., Bamshad, M. J., & Tabor, H. K. (2014). Attitudes of non-African American focus group participants toward return of results from exome and whole genome sequencing. *American Journal of Medical Genetics Part A*, *164*(9), 2153–2160. 10.1002/ajmg.a.36610 PMC4357389 24845082

[CIT0036] Yu, J. H., Crouch, J., Jamal, S. M., Tabor, H. K., & Bamshad, M. J. (2013). Attitudes of African Americans toward return of results from exome and whole genome sequencing. *American Journal of Medical Genetics Part A*, *161*(5), 1064–1072. 10.1002/ajmg.a.35914 PMC3635144 23610051

[CIT0037] Yu, J. H., Harrell, T. M., Jamal, S. M., Tabor, H. K., & Bamshad, M. J. (2014). Attitudes of genetics professionals toward the return of incidental results from exome and whole-genome sequencing. *The American Journal of Human Genetics*, *95*(1), 77–84. 10.1016/j.ajhg.2014.06.004 24975944 PMC4085580

